# Mitoprotective Effects of *Centella asiatica* (L.) Urb.: Anti-Inflammatory and Neuroprotective Opportunities in Neurodegenerative Disease

**DOI:** 10.3389/fphar.2021.687935

**Published:** 2021-06-29

**Authors:** Jia Hui Wong, Anna M. Barron, Jafri Malin Abdullah

**Affiliations:** ^1^ Neurobiology of Aging and Disease Laboratory, Lee Kong Chian School of Medicine, Nanyang Technological University Singapore, Singapore; ^2^ Department of Neurosciences, School of Medical Sciences, Universiti Sains Malaysia, Kota Bharu, Malaysia; ^3^ Brain & Behaviour Cluster and Department of Neurosciences, School of Medical Sciences, Universiti Sains Malaysia, Kota Bharu, Malaysia

**Keywords:** medicinal plants, neuroprotection, mitochondria, neurodegeneration, *centella asiatica* (L.) Urb, antioxidative, mitoprotective

## Abstract

Natural products remain a crucial source of drug discovery for accessible and affordable solutions for healthy aging. *Centella asiatica* (L.) Urb. (CA) is an important medicinal plant with a wide range of ethnomedicinal uses. Past *in vivo* and *in vitro* studies have shown that the plant extract and its key components, such as asiatic acid, asiaticoside, madecassic acid and madecassoside, exhibit a range of anti-inflammatory, neuroprotective, and cognitive benefits mechanistically linked to mitoprotective and antioxidant properties of the plant. Mitochondrial dysfunction and oxidative stress are key drivers of aging and neurodegenerative disease, including Alzheimer’s disease and Parkinson’s disease. Here we appraise the growing body of evidence that the mitoprotective and antioxidative effects of CA may potentially be harnessed for the treatment of brain aging and neurodegenerative disease.

## Introduction

*Centella asiatica* (L.) Urb. (CA) is a medicinal plant commonly consumed in salads or juices in several countries, including Malaysia, India, Sri Lanka, Indonesia and China (Hashim, 2011; Maulidiani et al., 2012; Bachok et al., 2014; Singh et al., 2014). CA has a wide range of ethnomedical applications, including treatment of gastrointestinal disorders, skin diseases, fever, and cognitive and memory problems (Gohil et al., 2010; Jahan et al., 2012; Sabaragamuwa et al., 2018). Studies of the plant extract and its bioactive compounds have revealed a broad range of pharmacological and therapeutic effects, including anti-ulcer (Zheng et al., 2016), anti-microbial (Idris and Nadzir, 2017), cytoprotective (Choi et al., 2016; Tewari et al., 2016), anti-inflammatory (Choi et al., 2016; Park et al., 2017; Ho et al., 2018), anti-oxidant (Zhao et al., 2014; Dewi and Maryani, 2015; Intararuchikul et al., 2019) and mitoprotective (Gray et al., 2017; Zhang et al., 2017; Gray et al., 2018c) properties. The bioactive components of CA readily cross the blood brain barrier and exert beneficial neuroactive effects in a range of models of aging (Zweig et al., 2021) and neurodegenerative disease including Alzheimer’s disease (AD) (Gray et al., 2018c; Matthews et al., 2019) and Parkinson’s disease (PD) (Gopi and Arambakkam Janardhanam, 2017; Teerapattarakan et al., 2018). Recent studies have associated these neuroprotective and anti-inflammatory effects with increased expression of proteins essential for mitochondrial bioenergetics and antioxidant genes (Gray et al., 2018c; Lu et al., 2021; Zweig et al., 2021). Mitochondria play a pivotal role in aging and neurodegeneration, regulating energy metabolism, immune responses and cell death pathways (Moreira et al., 2010; Rizzuto et al., 2012; Mills and O'Neill, 2016; Sun et al., 2016; Shah et al., 2019). Hence, this review focuses on the potential therapeutic application of CA for the treatment of brain-aging and neurodegenerative disease through restoration of mitochondrial function and inhibition of oxidative damage.

## *Centella asiatica* (L.) Urb. (CA): The Medicinal Plant

### Botany and Geographical Distribution of *Centella asiatica* (L.) Urb.

CA is commonly known by several names, including *gotu kola* in Sinhala, *pegaga* in Malay, ‘léi gōng gēn’ in Chinese and Asian or Indian Pennywort in English (Jahan et al., 2012; Orhan, 2012; Singh et al., 2014; Gajbhiye et al., 2016). CA belongs to the Apiaceae family, which is native to Asian countries and parts of China as well as several other parts of the world, such as northern Australia and the Western Pacific. The plant grows horizontally, with long, slender and tender prostrate stolons that can extend up to 2 m and are characterized by long internodes and nodes. Each node of the stem bears one to three leaves that are about 2–6 cm in length and 1.5–5 cm in width with a slightly cupped circular-reniform shape and palmately netted veins. CA is odorless and flowers from April to June with fascicled umbels that consist of three to four sessile flowers. These flowers bear 4-mm-long fruits that range in shape from oval to globular. Found up to 1800 m above sea level, CA grows in a wide range of habitats, such as open sunny areas, swamps, paddy fields as well as along the banks of lakes and ponds and on stone walls and rocks (Roy et al., 2013; Singh et al., 2014; Sirichoat et al., 2015; Gajbhiye et al., 2016).

### *Centella asiatica* (L.) Urb. and its Major Phytochemical Constituents

**FIGURE 1:**
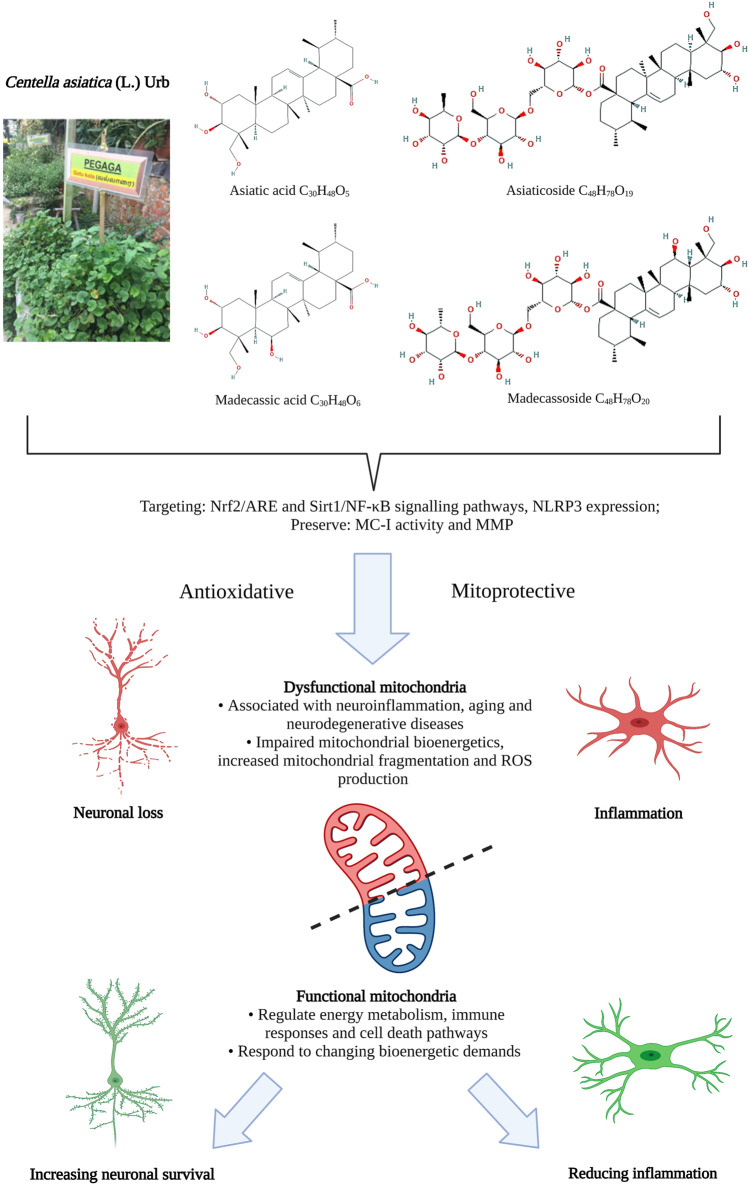
Antioxidative and mitoprotective activities of *Centella asiatica* and its main components. Mitochondrial dysfunction in regulating energy metabolism in response to changing bioenergy demands is closely associated with neuroinflammation in aging and neurodegenerative diseases. The antioxidative and mitoprotective activities of CA targeting mitochondrial and oxidative functions may confer neuroprotective benefits that could potentially be harnessed to treat aging and neurodegenerative diseases and improve functional behavioral outcomes. ARE, antioxidant response element genes; MC-I, mitochondrial complex I; MMP, mitochondrial membrane potential; NF-κB, nuclear factor kappa-light-chain-enhancer of activated B cells; NLRP3, NLR family pyrin domain containing three; Nrf2, NF-E2-p45-related factor 2; Sirt1, Sirtuin 1. Figure created with BioRender.com.

CA contains amino acids, alkaloids, carbohydrates, vitamins, minerals, terpenes of various categories (such as monoterpenes, sesquiterpenes, diterpenes, triterpenes and tetraterpene) and phenolic compounds (such as the flavonoids, tannins and other constituents). The phytochemistry of CA has previously been comprehensively reviewed by Brinkhaus et al. (2000), Gray et al. (2018a) and Torbati et al. (2021) therefore will only be summarized briefly here. Terpenes are the dominant group of chemical constituents of CA, with triterpenes being the major and most important component of CA, serving as a marker constituent for quality control analyses (Rafi et al., 2018). The triterpenes (Figure 1) found in CA are mostly pentacyclic triterpenic acids (sapogenins), such as the asiatic acid (PubChem CID: 119034, National Center for Biotechnology Information, 2021a) and madecassic acid (PubChem CID: 73412, National Center for Biotechnology Information, 2021b), and their respective triterpenoid glycosides (saponins, with a trisaccharide moiety linked to the aglycones), such as asiaticoside (PubChem CID: 52912190, National Center for Biotechnology Information, 2021c) and madecassoside (PubChem CID: 131801373, National Center for Biotechnology Information, 2021d) (Azerad, 2016; Rafi et al., 2018).

CA extract has been widely studied in the form of ethanolic (Sari et al., 2014; Sari and Rochmah, 2015; Binti Mohd Yusuf Yeo et al., 2018; Suri et al., 2018; Wong et al., 2019; Wong et al., 2020), methanolic (Veerendra Kumar and Gupta, 2003; Arora et al., 2018) and aqueous (Mitha et al., 2016; Gray et al., 2018c; Chintapanti et al., 2018) extract as well as leaf juice (Rao et al., 2007; Thirawarapan et al., 2019). Of these different preparations of CA, it was found that the ethanolic extract retained the highest amount of the triterpenes asiatic acid and asiaticoside compared to other solvents (Puttarak and Panichayupakaranant, 2013; Gajbhiye et al., 2016).

Wide chemotypic variations in triterpenoids were found in CA planted in different growing regions, altitudes and localities (Long et al., 2012; Singh et al., 2014; Srivastava et al., 2014). Genotypic and phenotypic variability have been associated with differences in phytochemicals content of CA including macronutrients, micronutrients, phenolics, flavonoids, tannin, anthocyanin, carotenoids and ascorbic acid (Thomas et al., 2010; Singh et al., 2014; Lal et al., 2017; Chandrasekara et al., 2020). Other than geographical and genotypical influences, the phytochemical compositions of CA also vary due to seasonal variations associated with the cultivation and harvesting procedures, light conditions, as well as the drying conditions post-harvesting (Maulidiani et al., 2012; Rahajanirina et al., 2012; Alqahtani et al., 2015; Plengmuankhae and Tantitadapitak, 2015). This underlines the potential challenges involved in the study of CA plant extract, as differences in specific phytochemical composition may influence the efficacy of the extract.

### Neuroactive Effects of *Centella asiatica* (L.) Urb.: Crossing the Blood Brain Barrier

Several pharmacokinetic studies have confirmed that bioactive components of CA can cross the blood brain barrier (BBB) when administered peripherally, although the transport mechanisms of these phytochemicals remain largely unknown. For example, asiatic acid, asiaticoside and madecassoside were found to accumulate in the brains of animals administered with CA extract or the respective single components (Yin et al., 2012; Anukunwithaya et al., 2017a; Anukunwithaya et al., 2017b). A recent study using primary porcine brain endothelial cells as *in vitro* BBB model also reported that asiatic acid, asiaticoside and madecassoside exhibit high permeability across the BBB (Hanapi et al., 2021). The bioavailability of these phytochemicals in brain tissue after peripheral administration (Yin et al., 2012; Anukunwithaya et al., 2017a; Anukunwithaya et al., 2017b) indicates they cross the BBB at adequate concentrations to exert neuroactive effects supporting the potential use of these compounds as neurotherapeutics.

### Neuroactive Effects of *Centella asiatica* (L.) Urb.: Cognition

Cognitive-enhancing effects of CA extract have been described in numerous studies, in both normal animals and models of aging and neurodegenerative disease (Doknark et al., 2014; Sari et al., 2014; Sirichoat et al., 2015; Yolanda et al., 2015; Wong et al., 2019; Sbrini et al., 2020). In early studies, CA extract was found to improve memory and ameliorate biochemical and mitochondrial dysfunction in a mouse model of aging (Kumar et al., 2011). In other studies CA was found to confer protection against hippocampal dysfunction, a region of the brain that plays a critical role in learning and memory and is severely affected in AD (Veerendra Kumar and Gupta, 2003; Giribabu et al., 2014). Further, key bioactive components of CA have also been shown to affect learning and memory in models of aging and neurodegenerative disease. For example, asiaticoside has been found to enhance cognitive performance in aged animals (Lin et al., 2013) and a rat model of AD (Zhang et al., 2017). The cognitive effects of CA extract have been linked to changes in synaptic plasticity (Lin et al., 2013) and excitatory neurotransmission (Wanasuntronwong et al., 2018; Wong et al., 2020) as well as improved neuronal health and survival in models of aging and disease (Gray et al., 2018b; Gray et al., 2018c). Here we will examine the evidence that CA and its phytochemicals provide cognitive benefits in aging and neurodegenerative disease *via* mitoprotective and antioxidant mechanisms (Soumyanath et al., 2012; Chen et al., 2016; Gray et al., 2016; Gray et al., 2017; Matthews et al., 2019).

## Targeting Mitochondria in Aging and Neurodegenerative Disease: Role for *Centella asiatica* (L.) Urb.

Mitochondrial dysfunction is closely associated with aging (López-Otín et al., 2013; Sun et al., 2016), AD (Moreira et al., 2010; Yoo et al., 2020) and PD (Yang et al., 2020). Mitochondria regulate energy metabolism, immune responses and cell-death pathways through their highly flexible and dynamic network. The mitochondrial network responds to changing bioenergetic demands by adjusting the rate of mitochondrial fission and fusion—a function that was found to be affected in most age-associated neurodegenerative conditions (Shah et al., 2019). Studies have shown that age-related toxic protein aggregates, such as Alzheimer’s beta amyloid (Aβ), induce mitochondrial dysregulation by binding to mitochondrial proteins. For example, Aβ has been found to bind to the mitochondrial fission protein (Drp1), and the mitochondrial voltage-dependent anion channel (VDAC) (Manczak et al., 2011; Manczak et al., 2018). These abnormal protein interactions affect mitochondrial biogenesis, increase mitochondrial fragmentation and induce free radical production (John and Reddy, 2020).

Mitochondria are the primary source of free radicals, otherwise known as reactive oxygen species (ROS), and ROS overproduction leads to oxidative damage. Oxidative damage further affects the mitochondrial respiratory chain function in generating energy in the form of adenosine triphosphate (ATP) through oxidative phosphorylation (OXPHOS) (Elfawy and Das, 2019). Perturbations in the electron transport chain function and/or reduction in the mitochondrial membrane potential lead to a vicious cycle of mitochondrial stress, which results in decreased ATP production and increased ROS production (Szalardy et al., 2015; Zorova et al., 2018). The brain is highly susceptible to both bioenergetic dysfunction and oxidative damage due to the high energy demands associated with neurotransmission and a high lipid content, respectively. The use of antioxidant strategies has been reported to provide a protective benefit against aging and neurodegenerative diseases. Further, enhancing mitochondrial biogenesis and quality control may be an efficient strategy for preventing mitochondrial disorders (Smith et al., 2012; Suliman and Piantadosi, 2016; Murphy and Hartley, 2018) and providing neuroprotection in AD and PD mouse models (Johri and Beal, 2012). Several therapeutic approaches that aim to protect against neurodegeneration and inflammation by improving brain bioenergetics, rescuing mitochondrial dysfunction and reducing oxidative damage are being developed (Cunnane et al., 2020; Fairley et al., 2021). In this section, we will focus on the mitoprotective and antioxidative effects of CA and its key phytochemicals as potential therapeutic agents that can 1) promote neuronal health and survival, and 2) reduce neuroinflammation.

### Neuroprotective Effects of *Centella asiatica* (L.) Urb. and its Major Constituents: Antioxidative and Mitoprotective Effects

Neuroprotective effects of CA have been described in several models of neurodegenerative disease and injury, linked to effects on mitochondrial energy production, oxidative stress and mitochondrial-induced apoptosis. For example, the CA extract, asiatic acid has been shown to prevent mitochondrial morphology abnormalities in a rat model of kainic acid-induced seizure, which protected synaptic function and alleviated cognitive deficits (Lu et al., 2021). In a separate study, the CA phytochemical asiaticoside was found to inhibit Aβ-induced neuronal apoptosis by restoring and maintaining mitochondrial membrane potential (Song et al., 2018). Several potential molecular mechanisms mediating the mitoprotective effects of CA have been proposed, including increased conductance and stabilization of VDAC (Tewari et al., 2016). VDAC plays a critical role in cell survival, transport of substrates for energy production and maintenance of mitochondrial membrane potential (Camara et al., 2017), making it a target of interest in regulating mitochondrial function.

Meanwhile, other studies have implicated CA and its bioactive components in the regulation of important antioxidant response signaling pathways. In mouse models of AD, CA extract has been found to promote antioxidative responses, countering Aβ pathology-driven oxidative stress, mitigating neuronal loss around the plaques and improving memory function (Gray et al., 2017; Gray et al., 2018c). CA extract has also been found to protect rotenone-induced parkinsonism rats against lipid peroxidation, dopaminergic neuronal death and locomotor deficit. These protective effects were associated with increased antioxidant enzyme expression and preservation of mitochondrial complex I activity, which is responsible for the rate-limiting step in OXPHOS (Teerapattarakan et al., 2018). Madecassoside was also found to be effective at ameliorating the deficits observed in PD rat models *via* its antioxidative activities, maintaining the redox balance (Xu et al., 2013). Similarly, asiaticoside has been found to reduce oxidative stress induced by rotenone (Gopi and Arambakkam Janardhanam, 2017; Subaraja and Vanisree, 2019). Likewise, asiatic acid provided antioxidative benefits in a drosophila PD model, protecting mitochondria against rotenone-induced oxidative stress and apoptosis. The antioxidative properties of asiatic acid are also thought to mediate neuroprotection and improve spatial memory function in animals treated with valproic acid (Xu et al., 2012; Umka Welbat et al., 2016). Outside of the brain, antioxidative effects of CA are also observed in other organs and systems. For example, CA extract was found to inhibit lipid peroxidation in rotenone-treated rats (Intararuchikul et al., 2019) and regulate lipid metabolism *via* antioxidant effect (Zhao et al., 2014). These findings support the notion that the neuroprotective effects of CA and its bioactive components are at least in part mediated through enhanced antioxidative responses.

CA-induced antioxidative responses have been linked to the higher expression of antioxidant response element genes (AREs) activated *via* Nrf2 (NF-E2-p45-related factor two, encoded by the NFE2L2 gene) (Matthews et al., 2019). The Nrf2/ARE signaling cascade regulates a plethora of cellular activities, including metabolic reprogramming, mitochondrial physiology and biogenesis, antioxidant stress response, drug detoxification, inflammation, autophagy and unfolded protein response and proteostasis (Dinkova-Kostova and Abramov, 2015; He et al., 2020). Altered expression of Nrf2-targeted genes is associated with AD, and previous studies have demonstrated that the activation of Nrf2 ameliorates Aβ pathology and cognitive deficits in AD mouse models (Bahn et al., 2019). Consequently, activation of Nrf2 pathway represents a promising therapeutic direction for enhancing mitochondrial quality control and biogenesis in aging and neurodegenerative diseases (Kerr et al., 2017; Gureev et al., 2019; Gureev and Popov, 2019; Brandes and Gray, 2020; Bento-Pereira and Dinkova-Kostova, 2021). Subsequent studies found that Nrf2 is a crucial component of the mitoprotective effects of CA, whereby long-term CA treatment improved the cognitive performance of wild type but not Nrf2 deficient mice (Nrf2 knockout) (Zweig et al., 2021). Further, these studies associated hippocampal mitochondrial dysfunction with cognitive performance.

In addition to the general ability to induce antioxidant responses, disease-specific mitoprotective effects of CA have also been identified in models of PD. For example, CA components have been shown to block the translocation of α-synuclein to the mitochondria, therefore maintaining mitochondrial membrane integrity and ATP production (Ding et al., 2018). Further, pre-treatment with asiatic acid significantly decreased mitochondrial ROS production in a 1-methyl-4-phenyl-pyridine (MPP+)-induced neuroblastoma model of PD and protected the cells form the loss of mitochondrial membrane potential (Chen et al., 2019). Additionally, CA and its triterpenoids may also reduce ROS production (Gray et al., 2017; Nataraj et al., 2017), thus potentially restoring mitochondrial function in the central nervous system (Onyango et al., 2017). For example, madecassic acid inhibited ROS production in human retinal microvascular endothelial cells (hMRECs) following hypoxia-induced oxidative stress (Yang et al., 2016). The molecular targets mediating these effects are yet to be elucidated and whether they are generalized to other disease models remains to be determined.

### Anti-Inflammatory Effects of *Centella asiatica* (L.) Urb. and its Major Constituents

The mitochondrial and metabolic fitness of the brain’s innate immune system plays an important role in the neuroinflammatory responses involved in neurodegenerative diseases (Paolicelli and Angiari, 2019)—a concept known as “immunometabolism” (O'Neill et al., 2016). Mitochondrial-dependent OXPHOS and fatty acid oxidation (FAO) are associated with anti-inflammatory responses (Mills and O'Neill, 2016) while, on the other hand, inflammatory responses are associated with a shift toward non-mitochondrial erobic glycolysis (Rodríguez-Prados et al., 2010; Galván-Peña and O’Neill, 2014). This switch toward erobic glycolysis causes several functional changes: 1) rapid supply of ATP, 2) proinflammatory cytokine production, 3) rearrangement of the tricarboxylic acid (TCA) cycle and accumulation of intermediate metabolites, such as succinate and citrate, and 4) repurposing of the electron transport chain (ETC) to produce ROS (Lampropoulou et al., 2016; Millet et al., 2016; Mills et al., 2016). Furthermore, microglial activation releases neurotoxic factors, such as mitochondrial-generated ROS, that exacerbate the neuroinflammation, thus resulting in neuronal death and neurodegeneration (González et al., 2014; Simpson and Oliver, 2020). Microglia are metabolically plastic and, hence, are potential therapeutic targets for the treatment of AD using metabolic reprogramming strategies (Fairley et al., 2021).

CA and its derivatives have also been shown to affect inflammatory responses through the regulation of mitochondrial and oxidative functions. Asiatic acid, asiaticoside and madecassoside have been found to demonstrate anti-inflammatory effects through a reduction of cytokine levels and the activation of microglia in stroke models (Krishnamurthy et al., 2009; Chen et al., 2014; Luo et al., 2014). Sirtuin 1 (Sirt 1) protein is an important epigenetic regulator for many physiological processes, modulating downstream pathways by targeting proteins such as nuclear factor kappa-light-chain-enhancer of activated B cells (NF-κB) and plays a role in alleviating oxidative stress (Elibol and Kilic, 2018). In an immortalized microglial cell line, asiatic acid was found to prevent LPS-induced neuroinflammation by enhancing Sirt1 expression while suppressing NF-κB activation, attenuated the production of nitric oxide and the expression of inducible nitric oxide synthase (iNOS) and reduced the expression and release of inflammatory cytokines in response to LPS-induced inflammation (Qian et al., 2018). Asiatic acid was shown to protect BV2 cells from LPS-induced damage by suppressing NLRP3 (NLR family pyrin domain containing three) expression and decreasing mitochondrial ROS, effectively ameliorating mitochondrial dysfunction (Chen et al., 2019).

Anti-inflammatory effects have also been reported in models of AD. In a study that used the intracerebroventricular infusion of toxic forms of Alzheimer’s Aβ, the neuroprotective effects of asiaticoside in Aβ-infused rats were suggested as being associated with the anti-inflammatory properties of asiaticoside, hence mitigating mitochondrial injuries and regulating the expression of apoptosis markers (Zhang et al., 2017). The mitoprotective effects of asiatic acid have been demonstrated in earlier studies that targeted the regulation of the mitochondrial membrane potential and ROS production (Xiong et al., 2009; Xu et al., 2012). Taken together, these findings demonstrate that CA and its major phytochemicals inhibit ROS production and ameliorate mitochondrial dysfunction, reducing detrimental inflammatory responses.

## Conclusion

Plants produce chemically, structurally and molecularly diverse phytochemicals that determine their evolutionary success. These compounds represent biological functions and continue to provide crucial novel pharmacological leads for the treatment of human diseases. CA and its phytochemicals have wide ethnopharmacological applications in various cultures, and its biological effects have been substantiated in numerous studies. These findings suggest that CA confers pleiotropic neuroprotective and anti-inflammatory benefits through its mitoprotective and antioxidative effects, which could potentially be harnessed for the treatment of aging and neurodegenerative diseases. Further research is still needed to determine the synergistic effects, safety, efficacy, bioavailability and metabolism of these components.
